# Pomegranate Fruit Growth and Skin Characteristics in Hot and Dry Climate

**DOI:** 10.3389/fpls.2021.725479

**Published:** 2021-08-19

**Authors:** Mukul Joshi, Ze’ev Schmilovitch, Idit Ginzberg

**Affiliations:** ^1^Institute of Plant Sciences, Agricultural Research Organization, Volcani Institute, Rishon LeZion, Israel; ^2^Institute of Agricultural Engineering, Agricultural Research Organization, Volcani Institute, Rishon LeZion, Israel

**Keywords:** epidermal density, fruit growth rate, hot climate, elastic modulus, pomegranate skin

## Abstract

Pomegranate (*Punica granatum* L.) fruit is well known for its health-beneficial metabolites. The pomegranate peel consists of an inner thick spongy white tissue, and an outer smooth skin layer that accumulates anthocyanins in red cultivars when ripe. The skin is made up of epidermis cells covered by a cuticle, the latter being the first target of cracking and russeting. The present study focuses on the effect of Israel’s hot and dry climate on pomegranate growth, to elucidate the derived effects on fruit skin characteristics and its putative resistance to the building pressure from fruit expansion. Experiments were conducted for four years, in four orchards located in different regions of the country, each with a different typical microclimate. Fruit-growth parameters were followed using remote-sensing tools, microscopic study, and mineral analysis of the skin, followed by determination of the peel’s elastic modulus. Fruit expanded in two phases: a short rapid phase followed by a gradual phase with a sigmoidal growth-rate pattern. Extreme hot and dry climate during the period of maximal growth rate was associated with restricted growth and a high proportion of small-size fruit. Anatomical study indicated that the skin of mature pomegranate fruit is made up of epidermal cells that are relatively flat and spaced apart, and is expected to be less durable against internal pressure. In contrast, skin of early immature fruit has two layers of dense and rounded epidermis, and is expected to be more resistant to cracking. Tensile strength studies confirmed this trend—skin of mature fruit had a lower elastic modulus than young fruit. However, restrained growth due to extreme environmental cues may result in better resistance of the mature pomegranate fruit to cracking, and in better skin quality and appearance, albeit small fruits. On the other hand, temperate climate at the beginning of the growth period, which allows high growth rate and high daily shrinkage, leads to pomegranate skin disorders.

## Introduction

Pomegranate (*Punica granatum* L.) fruit is well known for its health-beneficial metabolites (Holland et al., 2009; Viuda-Martos et al., 2010; Mphahlele et al., 2014; Singh et al., 2019), and there is extensive demand for it in global markets. The main pomegranate cultivar in Israel is “Wonderful,” which blooms in the spring, around the beginning of May, and is harvested toward the end of October. Hence, the fruit develops during the hottest months of the year. In particular, the first stage of fruit expansion by cell division occurs during heat waves with extreme temperatures (>34°C).

Suboptimal climate conditions during fruit development may result with physiological skin disorders, such as cracking and russeting. After a short period of cell division, fruit growth consists in the enlargement of fruit cells (Azzi et al., 2015), mainly due to the accumulation of water resulting from the balance between incoming (phloem and xylem) and outgoing (transpiration) fluxes (Ho et al., 1987). Changing balance between these fluxes results in variations in fruit volume. When water loss occurs during the day, the fruit shrinks (an elastic and reversible adjustment) to maintain its positive turgor pressure (Lechaudel et al., 2007; Jones and Syvertsen, 2011), and then it re-expands at night. Fruit expansion during growth requires plastic adjustment, which is an irreversible deformation of the cell walls, and is a function of cell-wall extensibility and turgor pressure (Lockhart, 1965; Trinh et al., 2021). Because the spherical organ shape does not provide any directional bias (i.e., cell structural elements), the mechanical signals are limited to stress intensity, resulting in continuous cell expansion until ripening, which approaches the limit of epidermal strength—as observed in fleshy fruit (Azzi et al., 2015). When turgor is high and the maturing (senescing) peel weakens, cracking may develop (Knoche and Lang, 2017; Ginzberg and Stern, 2019). In pomegranate, cracking is induced by differences in growth rate between the fruit peel and flesh and the pressure imposed by the quickly expanding arils on the stretched peel (Singh et al., 2020). Extreme and changing temperatures may further affect incoming and outgoing water fluxes, adding more strain on the peel.

The peel of pomegranate fruit consists of an inner thick spongy white tissue (mesocarp/albedo), and an outer smooth skin layer (exocarp/flavedo) which in red cultivars, turns red when ripe (Teixeira da Silva et al., 2013). The skin is made up of epidermis cells covered by a cuticle. Symptoms of cracking and russeting in pomegranate develop first as tiny cracks in the cuticle, and cv. “Wonderful” appears to be more susceptible to this than other cultivars (Drogoudi et al., 2021). This could be due to the different timing of its fruit set and harvest—“Wonderful” is a late cultivar. Nevertheless, since cracking appears at the cuticle level, and the skin is the external tissue resisting the turgor pressure, this study is focused on characterizing pomegranate skin and exploring the growth and climate factors that might affect its integrity; hereafter, “peel” refers to the skin together with the spongy tissue.

The present study focuses on the effect of Israel’s hot and dry climate on pomegranate growth, and elucidates the derived effects on fruit skin characteristics, and on the skin’s putative resistance to the pressure built up by fruit expansion. Experiments were conducted for four years, in four orchards located in different regions of the country, each with a different typical microclimate. We used remote-sensing tools to follow fruit-growth parameters, along with a microscopic study and mineral analysis of the skin, followed by a determination of the peel’s elastic modulus.

Fruit skin has dual role, it protects the fruit from environmental stresses, and at the same time, plays a critical role in resisting the internal growth pressures, controlling fruit expansion and maintaining fruit integrity. From an agricultural and commercial point of view, fruit skin disorders increase fruit waste and reduce yield, negatively affect fruit quality during storage, and negatively impact fruit appearance and marketability. Thus, a description of pomegranate fruit under various climate conditions could serve to plan orchard practices for the benefit of growers and consumers.

## Materials and Methods

### Plant Material and Experimental Design

Pomegranate fruit cv. “Wonderful” were collected from four commercial orchards, listed here according to their location from southern to northern Israel: orchard S, Shikma Field Crops, located at Kibbutz Mishmar HaNegev at the northern fringe of the Negev desert (31°22′55.5″N 34°43′00.8″E); orchard H, located at Kibbutz Hatzor, in the coastal plain area (31°4′526.6″N 34°42′49.9″E); orchard T, located at Kibbutz Tsor’a in the central region of Israel (31°45′37″N 34°57′48″E); orchard G, Woodland Hills (2002) ACS Ltd., located in Kibbutz Givat Haim (Ihud), the Hefer Valley region of the Sharon plain (32°23′46.3″N 34°57′32.0″E).

The experiments were conducted for 4 years: 2014—orchard H; 2015—orchards H, S, and G; 2016—orchards T, S, and G; 2017—orchards T and S. During the experimental years 2014–2017, and additional 2 years 2018–2019, data on commercial fruit from orchards T and S were collected from the packing factory; overall data on commercial fruit was collected for 6 years.

**FIGURE 1:**
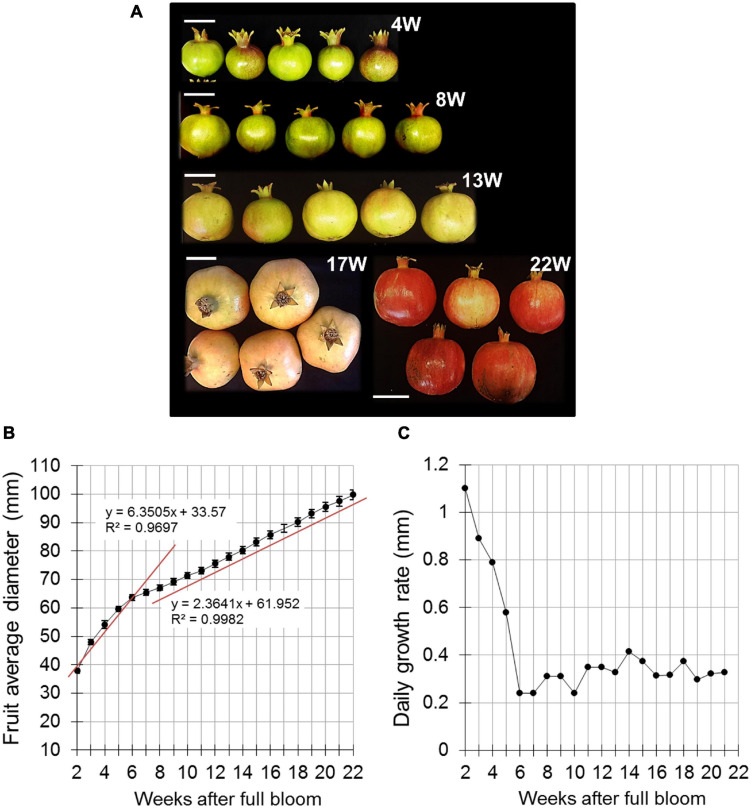
Pomegranate fruit size and growth rate during fruit development in 2014. **(A)** Presentation of fruit developmental stages indicated as weeks after full bloom (W). Bar = 5 cm. **(B)** Fruit diameter was measured manually using a caliper. Data represent an average of 20 fruit with ± SE. Trend lines with *R*^2^-value were added separately to the first and second growth phases (4–6 and 7–22 weeks after full bloom, respectively). **(C)** Daily growth rate was calculated based on the data presented in **(B)**.

For peel and skin analyses, fruit were collected every 2–3 weeks starting at 4 weeks after full bloom (WAFB) (Figure 1A), in 3–10 biological replicates from separate plots in the orchard.

### Fruit Measurements

Fruit size was measured manually in 2014 in orchard H with a caliper. The equatorial diameter of 20 labeled fruits that were randomly scattered throughout the orchard was measured every 2 weeks, starting at 2 WAFB until the end of growth at 22 WAFB.

**FIGURE 2:**
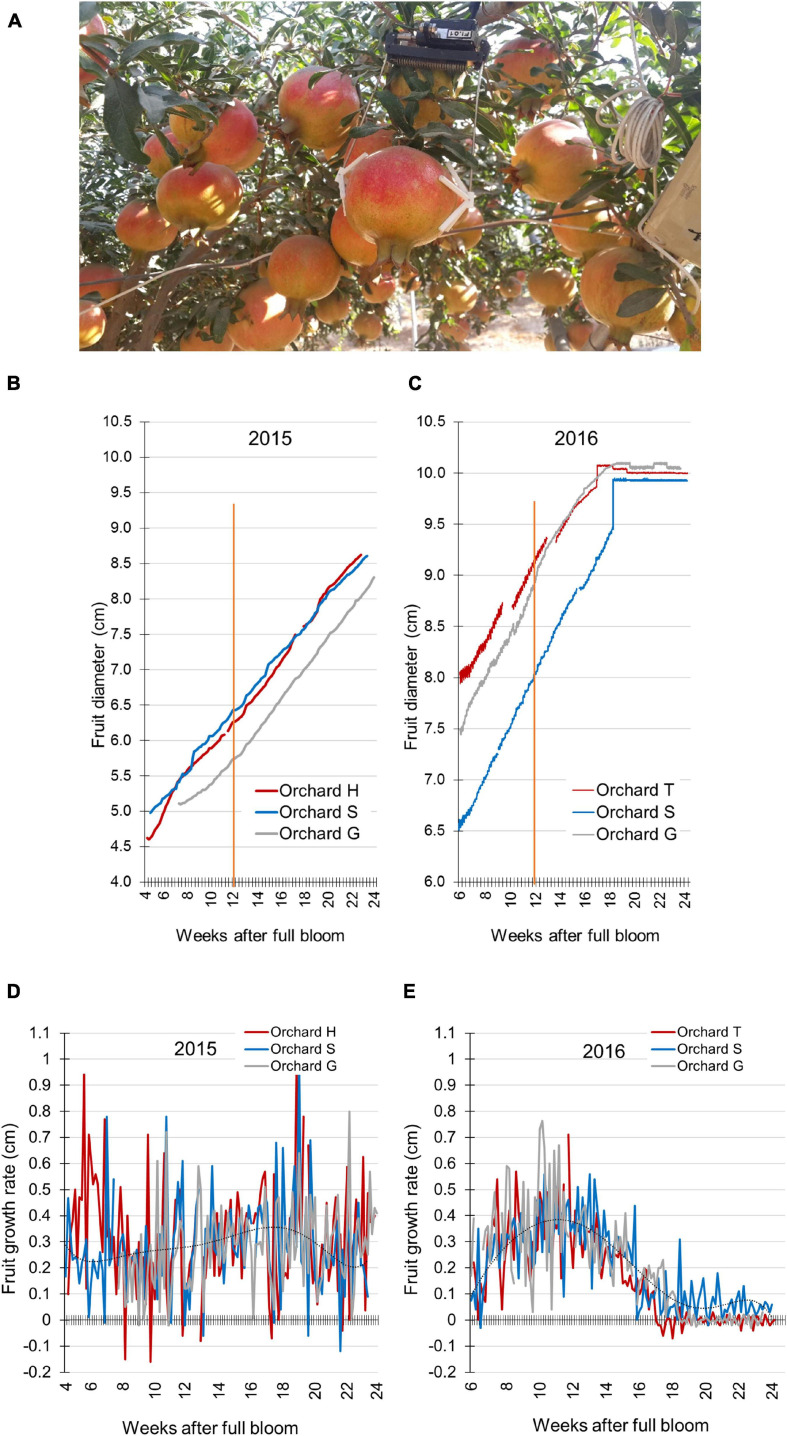
Pomegranate fruit size and growth rate monitored by the Phytech system. **(A)** Illustration of Phytech sensor and clamps. **(B,C)** Fruit diameter measured in orchards H, S, and G during 2015 **(B)** and in orchards T, S, and G during 2016 **(C)**. Orange line indicates mid-growth (12 weeks after full bloom). **(D,E)** Daily growth rate was calculated based on the data presented in **(B,C)**, respectively. A trend line was added on each chart to illustrate growth-rate pattern.

In the following years, fruit size was monitored using the Phytech sensor-based system (Phytech Ltd., Rosh Haayin, Israel).^1^ The system is usually used by growers to optimize production by monitoring plant water stress and analyzing climate data. Here we used the system for continuous monitoring of fruit expansion during development, by placing two plastic paws attached to a sensor on either side of the fruit at its equatorial plane (Figure 2A). This unit (sensor and paws) can monitor microvariations in fruit diameter, and the data were transmitted in real time to the Phytech cloud for storage until analysis. The Phytech system was used in 2 successive years (2015 and 2016), three orchards per year, with five sensor units in each orchard on selected fruit. The fruit were positioned under the tree canopy and not at its perimeter (Figure 2A).

Fruit diameter data were collected every hour during growth, and were used to determine daily fruit-growth rate by calculating the difference in fruit size when compared to the previous day at noon (12:00 h). Maximum daily shrinkage (*mds*) of the fruit was calculated by Phytech’s patented algorithm that quantifies the difference between maximal and minimal daily fruit diameter.

Climate data—temperature and humidity—under the canopy were also measured by the Phytech system. Climate data for the entire region in which the orchards were located, for the years 2015–2019, were obtained from the meteorological services of the Ministry of Agriculture and Rural Development, Israel.

### Pomegranate Skin Anatomical Study

Two approaches were used to study pomegranate skin anatomy: scanning electron microscopy (SEM) and light microscopy.

Sample preparation for SEM analysis: tissue blocks (4 mm × 3 mm × 3 mm) were sampled from the surface of the pomegranate fruit. These were fixed in FAA (50% ethanol, 5% acetic acid and 10% formaldehyde, v/v, in water), and then dehydrated in serial dilutions of ethanol. Samples were dried in a K850 critical point dryer (Quorum Technology Ltd., United Kingdom), then coated with gold-palladium alloy using an SC7620 mini sputter coater (Quorum Technology). The samples were analyzed by benchtop SEM, model JCM-600 (JEOL, Japan).

Sample preparation for light microscopy: tissue blocks from the surface of the pomegranate fruit (4 mm × 3 mm × 3 mm) were fixed in FAA (50% ethanol, 5% acetic acid, and 3.7% formaldehyde, v/v, in water), dehydrated in an ethanol/Histoclear series and embedded in paraplast (Surgipath Paraplast Plus, Leica Biosystems Richmond Inc., United States) according to standard methods (Ruzin, 1999). Tissue sections (20 μm) were stained with Safranin-O/Fast green (Sigma Chemicals, Israel) for morphological examination (Johansen, 1940). Sections were observed under a light microscope (Leica DMLB, Germany) and images were displayed on a monitor through a CCD camera (Leica DC2000) using the Leica IM1000 program.

The anatomical study was conducted with fruit collected every 3 weeks during fruit development in 2015, 2016, and 2017 from orchards S and T. Each sampling included peels from three independent fruit.

The same peel sections were used to measure epidermal cell density in the fruit skin. One cross section was selected from each fruit, and the number of epidermal cells was counted along an arbitrary 1-mm long line using the ImageJ program.^2^ This was done at three locations in the section, and the average cell number per 1 mm was calculated for each section. An average of three sections from three independent fruits yielded the cell density for each sampling.

### Quantification of Cuticle Amount

Fruit were collected at several time points during growth, in three replicates, each consisting of one fruit. Fruit skin was sampled using a cork borer as thin discs (1.5 cm in diameter), 13–15 discs per fruit. Skin discs were incubated in 0.1 M sodium acetate buffer (pH 3.8) for 1 week at 37°C with shaking at 30–50 rpm. The sodium acetate buffer was replaced with lysis solution containing 4.73 units/ml pectinase (Sigma Chemicals Israel, Cat. #17389) and 1.57 units/ml cellulase (Sigma Chemicals Israel, Cat. #C1184) in 0.1 M sodium acetate buffer, to remove epidermal and other cell components adhering to the cuticle membrane. Discs were incubated in the lysis solution for 2 weeks with replacement with fresh solution after 1 week. Purified cuticle membranes were dried in an oven at 40°C and weighed. Cuticle weight was quantified per square centimeter area of fruit surface and an average was calculated for the three replicates at each time point.

### Mineral Determination

Samples of pomegranate skin (only the colored layer, without the spongy tissue) were rinsed with deionized water, dried in an oven at 60°C and pulverized. Total concentrations of nitrogen (N), phosphorus (P), and potassium (K) were determined following digestion with sulfuric acid and peroxide (Snell and Snell, 1949), and of calcium (Ca) and magnesium (Mg) following digestion with nitric acid and perchlorate. N and P were determined using an Autoanalyzer (Lachat Instruments, Milwaukee, WI), and K, Ca, and Mg were determined by atomic absorption spectrophotometer (Perkin-Elmer 460).

### Pomegranate Peel Elasticity

Fruit were sampled from orchards T and S every 3 weeks during the growth period in 2016 and 2017. For technical reasons, the whole peel—colored skin and spongy tissue—was used for this analysis. Peel strips were sampled from 5 to 10 fruits (depending on fruit size) at each time point; two strips along the vertical axis of the fruit and two parallel to the equatorial axis. The peel samples were cut out using a plastic mold puncturer with sharp edges into strips of 6 cm length and 2 cm width edges, with a 2-cm long middle region of 1 cm width. Immediately after shaping the strips, peel thickness was measured using a caliper, and the strip was attached to clamps of a Universal Testing Machine (LRX; Lloyd Instruments, United Kingdom) (Hetzroni et al., 2011). Strips were subjected to tensile loading with a crosshead speed of 10 mm/min until rupture point. Data were then analyzed using NEXYGEN (v 4.1) software. Tensile strength (MPa) was calculated by dividing the “extension at break” value (N) by the cross-sectional area (thickness × width) at the middle region of the strip. “Apparent elastic modulus” (E) was calculated using the extension at break values multiplied by the length of the middle region of the strip (2 cm), and divided by the tensile strength.

### Data Analysis

Data were analyzed for statistical significance by Student’s *t*-test using JMP software.^3^ Significant difference was determined at *P* < 0.05.

## Results

### Fruit Growth

A preliminary experiment was conducted in 2014 to monitor fruit growth in orchard H. Equatorial diameter of the fruit was measured manually every day, starting from 2 WAFB; respective developmental stages are given in Figure 1A. Data showed increasing fruit expansion until 22 WAFB (harvest was about 2 weeks later) (Figure 1B). This was divided into two growth phases—from 2 to 6 WAFB, the calculated slope representing the average rate of fruit diameter increase was sharper than that for the period of 7–22 WAFB (Figure 1B). Accordingly, calculation of daily growth rate indicated a higher rate from 2 to 6 WAFB compared to the following growth period (Figure 1C). However, the growth rate of the first period declined sharply, whereas it remained low, albeit slightly fluctuating with a peak at 14 WAFB, in the growth period that followed (7–22 WAFB).

The following years, fruit growth parameters were monitored in several orchards using the Phytech system. This included orchards H, S, and G in 2015, and orchards T, S, and G in 2016. Due to technical limitations—size and spacing of the sensor clamps (Figure 2A)—measurements were initiated at around 4–6 WAFB, probably missing most of the first growth period seen in Figure 1B. A linear growth pattern was obtained for all orchards in both years (Figures 2B,C). Nevertheless, fruit in 2015 were smaller than those in 2016. In 2015, at mid-growth (12 WAFB), the diameter of the fruit from the three orchards was in the range of 5.7–6.4 cm, and at the end of the growth period (24 WAFB), fruit size was around 8.5 cm in diameter (Figure 2B). In 2016, the range of fruit diameter at mid-growth was 8.0–9.1 cm, and it reached 10 cm—the maximal capacity of the Phytech clamps—at around 18 WAFB, before the end of the growth period (Figure 2C).

The Phytech data related to fruit size—bigger fruit in 2016—were confirmed when fruit (around 500 tons) collected from orchards S and T were sorted at the packing factory (Supplementary Figure 1): 59% of the 2016 fruit from orchard S were heavier than 650 g, the optimal commercial size, compared to only 4% of the 2015 fruit, and 17% of 2017 fruit from that orchard (Supplementary Figure 1A). Similarly, in orchard T, the 2016 fruit were bigger than the 2017 fruit (Supplementary Figure 1B).

The Phytech data were further used to calculate daily fruit-growth rate for all orchards. No clear pattern was obtained for fruit in 2015 (Figure 2D), whereas the 2016 fruit showed a sigmoidal growth pattern (7–16 WAFB), with a high daily expansion rate that peaked at mid-growth (11–12 WAFB) (Figure 2E). Daily growth rate can be affected by the transport of water and solutes in and out of the fruit, causing its expansion or shrinkage. Extreme daily changes in fruit size can weaken the fruit skin and increase its susceptibility to cracking. Accordingly, the daily shrinkage of the fruit was calculated, i.e., the daily difference between maximal size at night and minimal size at noon (irrespective of fruit size). In both 2015 and 2016, the first 10 days of measurements demonstrated highest *mds* values compared to the following growing period (Supplementary Figure 2). In 2016, *mds* values were relatively high at 7–14 WAFB and moderate later on (Supplementary Figure 2B). When compared to the growth-rate data, this implied that high *mds* values were obtained during the period of highest growth rate (7–16 WAFB; Figure 2E). The positive association of *mds* and growth rate was further demonstrated by the fact that the first 10 days of *mds* measurements showed the highest *mds* values (6–7.5 WAFB; Supplementary Figure 2B). This short period is the end of the first fruit-growth period with maximal growth rate (2–6 WAFB; Figure 1B).

In 2015, daily shrinkage was moderate, and decreased slowly toward harvest time (6–24 WAFB; Supplementary Figure 2A). This is also in accordance with the stable growth rate of the fruit (Figure 2D).

As already noted, extreme daily changes in fruit size can weaken the fruit skin, increase its susceptibility to cracking, and reduce its marketability. This was demonstrated when fruit of orchard T were analyzed at the packing factory for export quality (total of 467 and 766 tons of fruit for 2015 and 2016, respectively). In 2015, the yield of high-quality fruit was 80%, of which 38% were of premium quality. In 2016, the yield of high-quality fruit was only 54.6%, of which 23.4% were of premium quality.

### The Effect of Local Climate on Fruit Growth

The different patterns of fruit size and growth rate between experimental years could result from different yearly and local climate conditions. In 2015, high temperatures (≥34°C) with occasional warmer heat waves (40°C) were measured in the orchards, especially from mid-July to mid-August (9–14 WAFB; Supplementary Figures 3A–C), the period with highest fruit-growth rate. Minimal temperatures during the night were occasionally high as well (>20°C). From mid-September, temperatures dropped in all orchards and percent relative humidity (%RH) increased. In 2016, at the beginning of the growing season, temperatures were around 35°C; however, from mid-August they declined gradually to 30°C, without any of the 40°C heat waves observed the year before (Supplementary Figures 3D–F). Overall, 2015 was hotter and with heat waves compared to 2016, especially during the developmental phase when growth rate was highest. This possibly explaining the reduced fruit size (Figure 2B) and uncharacteristic growth-rate pattern (Figure 2D).

To test the correlation between climate conditions and fruit size and quality, we analyzed the respective data of commercial plots collected by the packing factory (>2,000 tons of fruit) from orchards S and T for the years 2015 to 2019. Climate data were obtained from the meteorological services of the Ministry of Agriculture (Supplementary Figure 4), and were expressed as number of days during July–August of 2015–2019 for which the maximal temperature was above 34 or 40°C, and %RH was below 20, and as average minimum RH during July–August (Table 1, bottom). Note that orchard S is located in the Negev region of Israel, characterized by a hot and dry climate, compared to orchard T, which is located in the temperate region of the country.

**TABLE 1:** Multiyear data of fruit size and climatic conditions.

Fruit size group	Fruit weight (g)	Fruit percentage (%)*									
		Orchard S					Orchard T				
		Years					Years				
		2015**	2016	2017	2018	2019	2015**	2016	2017	2018	2019
A	1040+		2.9	0.1	3.8	4.4		2.3	1.3	3.9	4.3
B	950–1,040		4.0	0.4	11.5	8.9		3.3	3.0	9.9	8.2
C	885–950		7.9	1.3	12.6	12.6		5.6	5.6	13.0	13.4
D	750–850		21.0	5.6	20.0	13.8		21.9	15.2	23.8	17.6
E	655–750		14.6	9.7	15.7	19.5		15.6	15.1	17.4	19.7
F	510–655		27.7	31.5	22.2	26.5		30.1	32.4	24.4	27.1
G	410–510		14.1	28.7	8.9	9.0		15.4	19.2	6.3	7.8
H	365–410		4.0	11.8	3.1	2.7		3.5	5.2	1.0	1.5
I	305–365		2.7	7.1	1.1	1.6		1.9	2.2	0.2	0.4
J	255–305		0.8	2.9	0.7	0.7		0.3	0.4	0.0	0.1
K	225–255		0.2	0.7	0.2	0.2		0.0	0.0	0.0	0.0
H	200–225		0.1	0.2	0.1	0.0		0.0	0.5	0.0	0.0
Total fruit (%)^†^											
>650 g		4.3	50.5	17.1	63.6	59.3	n.a.	48.7	40.2	68.0	63.1
<650 g		93.4	49.5	82.9	36.4	40.7	n.a.	51.3	59.8	32.0	36.9

**Number of days with extreme high temperature during July–August**											

>34°C		47	44	55	43	50	25	7	31	10	20
>40°C		3		3	1	1					

**Dry conditions during July–August**											

Average minimal %RH		30.4	36	34.5	37.5	31.9	35.9	40.0	37.9	38.9	38.1
%RH ≤ 20^††^		10	0	4	2	8	5	0	1	1	5

*The percentage of fruit in each size group, out of the total yield for the specified year.

**Data from experimental plots.

*^†^Annual summary of the percentage of fruit bigger and smaller than 650 g.*

*^††^Number of days in which the %RH was equal to or lower than 20.*

In the region of orchard S, July–August of 2015 and 2017 were hotter than the same period in 2016, 2018, and 2019, with 47 and 55 days of high temperatures (>34°C), respectively, and with occasional heat waves above 40°C (Table 1, bottom). This was combined with dry conditions—low average minimum %RH and more days for which %RH was below 20 (Table 1, bottom). In these years, the percentage of big fruits (>650 g) was low (4.3% for 2015 and 17.1% for 2017), whereas in the years 2016 and 2018, which were not as hot or dry, over 50% of the fruit were bigger than 650 g (Table 1). Data from 2019 also showed hot and dry weather, similar to 2015 and 2017, with one short heat wave above 40°C; however, unexpectedly, over half of the fruit were big (Table 1). Examination of the temperatures during October, the last month of the growth period, may provide an explanation. During October of 2015 and 2017, the maximal temperature was steadily dropping to below 30°C; however, there were 4 days of temperature that was 4–9°C above the average maximal temperature (Supplementary Figure 4). In contrast, during October 2019, the temperature drop was steady, with only one peak of higher temperature. This suggests that stable temperatures (around 25–30°C) at the end of the growth period may have compensated for the growth-inhibition effect of the high temperatures in July–August 2019, resulting in big fruit. In 2015 and 2017, the temperature fluctuations in October did not allow for this compensation and the fruit remained small.

Similarly, for orchard T, 2015 and 2017 were hotter and drier during July–August compared to the other tested years (Table 1). There were no fruit-size data for this orchard for the year 2015, but in 2017, only 40% of the fruit were bigger than 650 g, whereas this percentage was much higher in 2016, 2018, and 2019 (Table 1). Although fruit load per tree may differ between orchards and years, which in turn affects fruit size, the overall data suggested an association between the development of small-size fruit (<650 g) and extreme hot and dry climate in the period of maximal fruit growth. Association analysis performed with data of both orchards supported this observation, indicating opposite trends for fruit size and number of days with high temperature >34°C, although the correlations were low (*R*^2^ = 0.45 for orchard S and 0.43 for orchard T; Supplementary Figure 5), and a high positive correlation for fruit size and minimal %RH for orchard S (*R*^2^ = 0.85), with no association for orchard T. This might imply that drought conditions, i.e., %RH < 35, during July–August also negatively affect fruit size.

### Skin Anatomy of Developing Pomegranate Fruit

**FIGURE 3:**
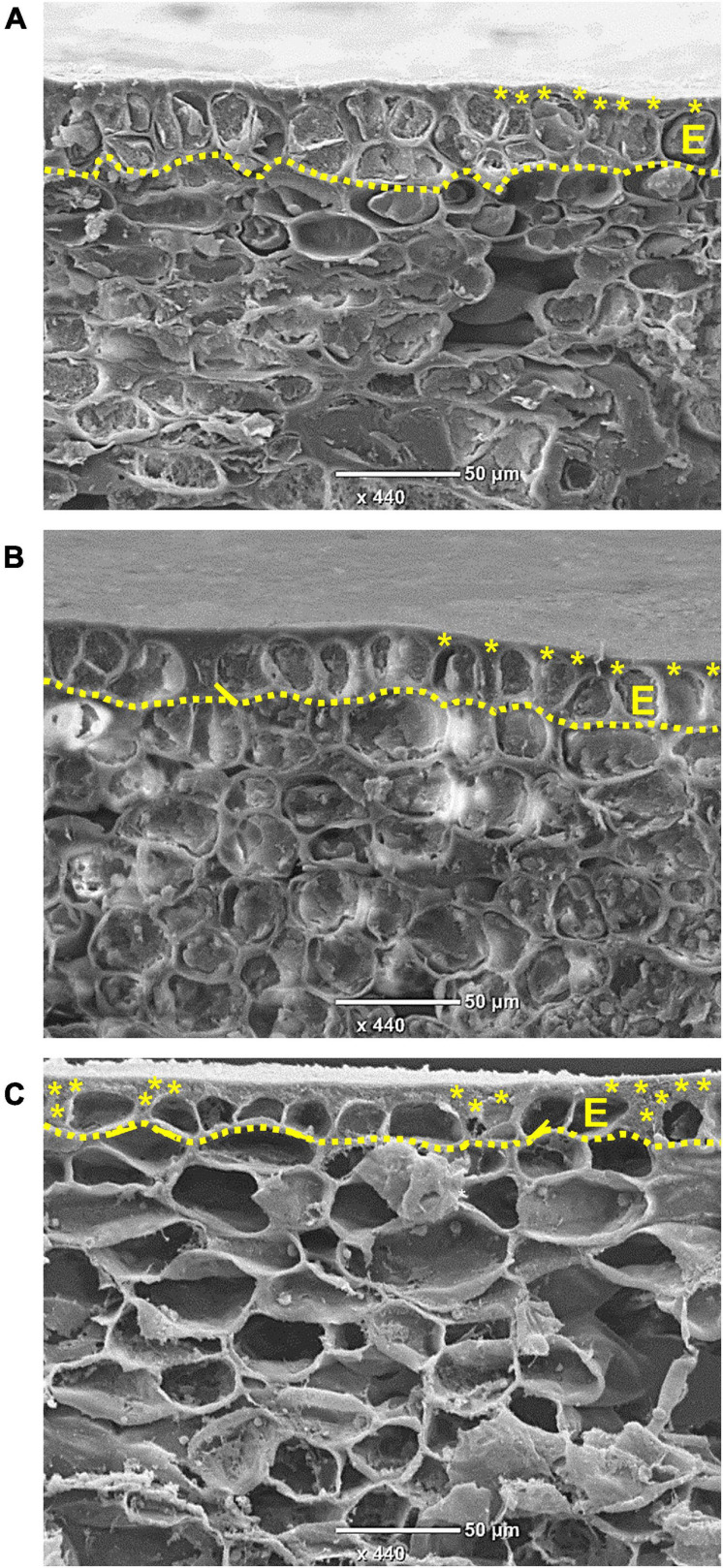
Pomegranate skin observed by scanning electron microscope. Micrographs were taken at 8 **(A)**, 11 **(B)**, and 24 **(C)** weeks after full bloom during the year 2014. Dotted line outlines the epidermal layer (E), and asterisks mark the cuticular layer and cuticular pegs between the cells. Bar = 50 μm.

**FIGURE 4:**
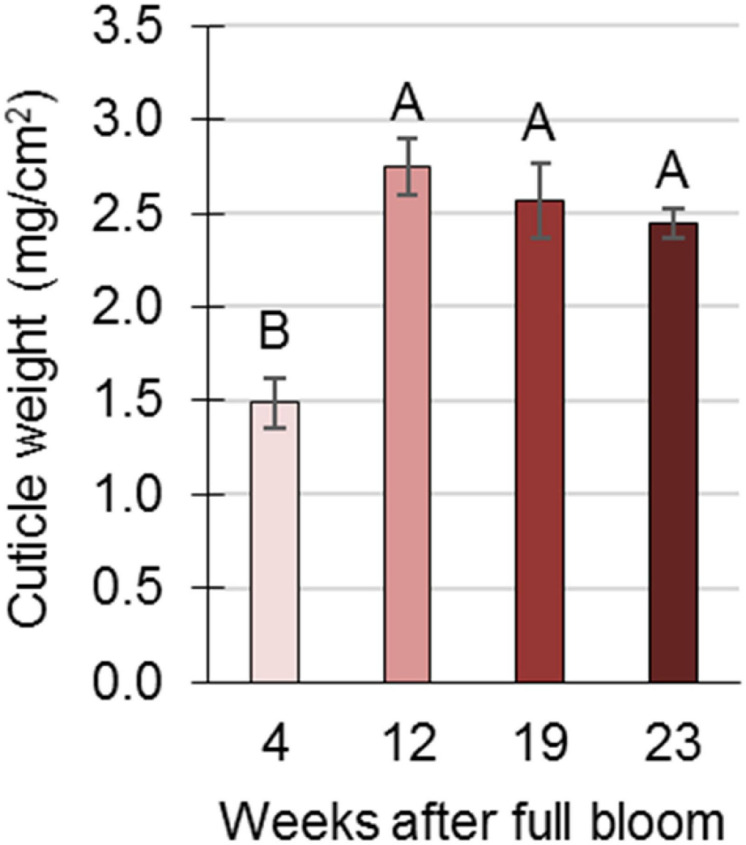
Cuticle determination in pomegranate fruit skin during development in 2014. Cuticle membrane was purified from underlying epidermal and parenchyma cells by enzymatic lysis in cellulose and pectinase solution and its weight (mg/cm^2^) was calculated as the average of three replicate fruits ± SE. Data were analyzed for statistical significance by Student’s *t*-test. Mean values with different letters differ significantly at *P* < 0.05.

An anatomical study of the skin during fruit development was conducted using SEM. In young fruit, at 8 WAFB, the epidermis consists of two cell layers, and an overlying cuticle (Figure 3A). As the fruit expands, at 11 WAFB, the epidermis is reduced to one cell layer (Figure 3B), and in mature fruit, at 24 WAFB, the epidermis layer is further “stretched”: the epidermal cells appear flattened compared to the more elongated cells at 11 WAFB, and the cuticular matrix occasionally fills the gaps between the cells (Figure 3C). Accordingly, a smaller amount of cuticle was found in the skin of early fruit (4 WAFB) compared to that of developing (12 WAFB) and maturing (19–23 WAFB) fruit, although the latter showed a slight but insignificant decrease in the amount of cuticle during maturation (Figure 4).

**FIGURE 5:**
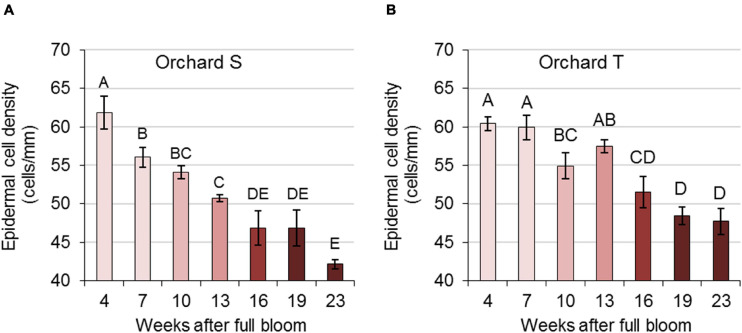
Epidermal cell density in pomegranate skin during the growing season of 2017. Data represent fruit from orchards S **(A)** and T **(B)**. The number of epidermal cells in histological sections was counted along a 1-mm line. Values are averages of three replicate fruit ± SE. Data were analyzed for statistical significance by Student’s *t*-test. Mean values with different letters differ significantly at *P* < 0.05.

To further determine epidermal cell density in the skin during pomegranate development, peel tissue was embedded in paraplast, sectioned and viewed under a light microscope (Supplementary Figure 6). The number of epidermal cells along an artificial 1-mm line of skin was counted. Data collected for orchards S and T during 2017 are presented in Figure 5, and data of previous years are presented in Supplementary Figure 7. In all experimental years and orchards, epidermal cell density was highest in young fruit, then decreased gradually and significantly during fruit development and expansion.

### Mineral Analysis of the Skin of Developing Pomegranate Fruit

**FIGURE 6:**
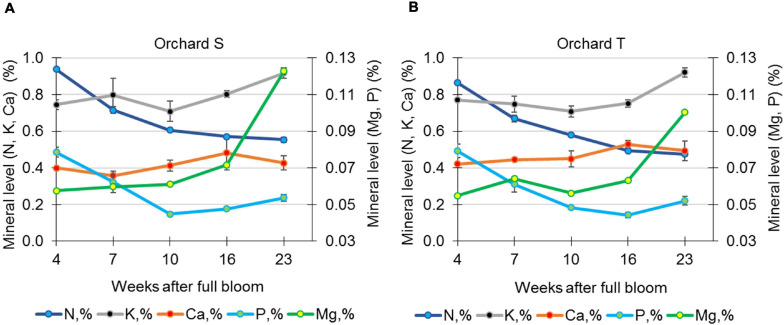
Determination of minerals in pomegranate skin during the growth season of 2017. Data represent fruit from orchards S **(A)** and T **(B)**. The concentrations of N, P, K, Ca, and Mg are given as percentage of skin tissue dry weight. Values are averages of three replicate fruits ± SE.

Mineral analysis of the skin during pomegranate development was performed in 2017 for fruit from orchards S and T. In the skin of fruit from orchard S, the level (percentage of dry matter) of N and P was high in early developing fruit (4 WAFB), then declined in the skin of mature fruit by 30–40% (23 WAFB, Figure 6). Mg showed the opposite pattern: its level in the skin of mature fruit was double that in young fruit, whereas K level stayed high with a slight increase (20%), and Ca level stayed the same throughout fruit growth. This pattern of mineral concentrations in the skin of developing fruit was similar for fruit collected in orchard T.

### Elasticity of Pomegranate Peel During Development

**FIGURE 7:**
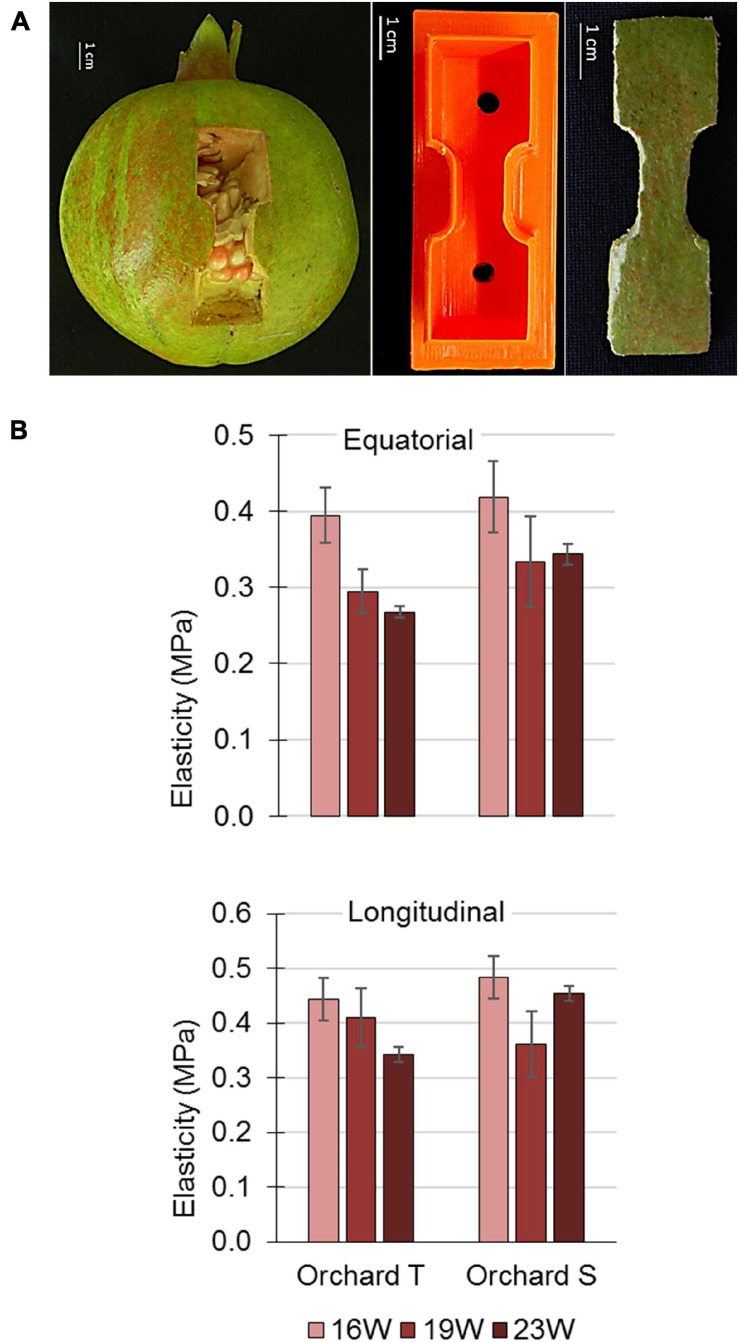
Elasticity of pomegranate peel during the second half of the 2017 growing season. **(A)** Illustration of longitudinal peel sampling for clamping in the Universal Testing Machine (left panel). For the experiment, two equatorial and two longitudinal peel strips were collected similarly from each fruit. The peel samples were shaped using a plastic mold (middle panel) with sharp edges into strips of 6 cm lengthwise and 2 cm width, with a 2-cm long middle region of 1 cm width (right panel). **(B)** Elasticity of fruit peel from orchards S and T. Data from each orchard and time point are an average of 5 fruit with ± SE.

The tensile strength of pomegranate peel was monitored during fruit growth, and peel elasticity was calculated as described in section “Materials and Methods.” Fruit peel was collected in the year 2015 from orchard S (data not shown), and in 2016 (Supplementary Figure 8) and 2017 (Figure 7) from orchards S and T. Peel samples were cut from the equatorial and longitudinal axes of the fruit (Figure 7A). Data of 2017 fruit from orchard T indicated reduced elasticity of the peel in mature fruit (23 WAFB) in comparison to an earlier developmental stage (16 WAFB), although the difference was not significant (Figure 7B). This difference applied to both the equatorial and longitudinal axes of the fruit. Data from orchard S showed similar non-significantly reduced elasticity only for the equatorial axis of the fruit (Figure 7B). Results from 2016 were not conclusive for fruit from orchard T. However, those for fruit from orchard S suggested an increasing trend in peel elasticity during the period of maximal growth rate, from mid-July to the end of August, 9–15 WAFB, with a slight, non-significant decrease at 22 WAFB when fruit growth subsides (Figure 2 and Supplementary Figure 8). At time points earlier than 9 WAFB, peel elasticity could not be monitored due to small fruit size, which did not allow excising a peel sample of the required size.

## Discussion

The nutritional value of pomegranate fruit is well known and consumer demand for the fruit and its juice is on the rise. When the fruit is marketed whole, its appearance is an important factor, especially in export markets. Optimally, in red cultivars, the skin is smooth and shiny, with an intense red color that covers the surface of the fruit evenly. However, non-optimal growth factors impair peel development and quality, and weaken its resistance to internal turgor pressure, resulting in russeting and cracking. The latter is not just an aesthetic defect; it reduces the final yield at harvest, as cracked fruit is considered waste. Here, we evaluated how the local hot and dry climate and its annual changes modify the characteristics of fruit growth and of its skin tissue.

### Pomegranate Fruit Growth and the Effect of Hot Climate

Fruit expansion, as measured by its diameter, exhibited two linear growth phases: up to 6 WAFB (mid-June), fruit-expansion rate was faster than in the following phase that extended to almost the end of the growth period (22 WAFB, 2014) (Figure 1). Linear expansion of the fruit was also measured in the subsequent experimental years (2015, 2016) in four orchards, using the Phytech system (Figures 2B,C), and followed a similar growth pattern of a rapid phase until mid-June and a gradual phase until harvest, as also observed previously (Ben-Arie et al., 1984). It has been suggested that the initial rapid increment in fruit growth occurs during cell division, which is characterized by growing kernel tissue, and increments in testa hardness (Shulman et al., 1984). Calculation of the daily growth rate indicated a single sigmoidal curve (Figure 2E; Ben-Arie et al., 1984; Shulman et al., 1984); however, in the hot and dry year of 2015, the characteristic sigmoidal growth-rate pattern flattened, and fruit were smaller than in 2016 (Figures 2B,D, Supplementary Figure 1A, and Table 1). These differences in growth pattern and fruit size were associated with extreme climate conditions during the period of maximal growth rate—mid-July to mid-August, 9–14 WAFB (Figures 2D,E and Supplementary Figure 3). This period in 2015 was characterized by extreme temperatures and low minimal %RH that extended for several days, compared to the same period in 2016, which was not as hot or dry (Table 1). This association was further demonstrated for the following years, 2017 and 2018: the climate of the former from mid-July to mid-August was hotter and dryer than the latter, and resulted in smaller fruit (Table 1 and Supplementary Figures 4, 5). Interestingly, the same association was not apparent in 2019; even though climate conditions were as hot and dry as in 2015 and 2017, most of the fruit were big. Comparison of climate conditions in those years suggested that a gradual decline in temperature (to around 25–30°C) in October 2019, the last month of growth, may have compensated for the growth-inhibition effect of the high temperatures during July–August, resulting in a high percentage of big fruit. In 2015 and 2017, fluctuations of high temperature in October prevented this compensation and fruit remained small (Table 1 and Supplementary Figure 4). Interactions of temperature, minimal %RH and fruit size (Supplementary Figure 5) suggested that a high number of days with %RH < 35 during July–August also negatively affects fruit size, implying that an extended dry climate also impairs fruit expansion. It was suggested that during periods of drought, fruit skin loses its ability to divide and enlarge (Singh et al., 2020). Conversely, high RH promotes fruit growth (Khadivi-Khub, 2015).

Fluctuations in maximum temperature and lower humidity have been positively correlated with pomegranate cracking at the end of growth (Drogoudi et al., 2021). In Israel, russeting due to microcracks develops at mid-growth around August-September, and may be related to the heat waves described above.

### Pomegranate Skin Characteristics—Anatomy, Mineral Content and Elasticity

Both the cuticle and the epidermal cells that produce it confer the skin/peel’s mechanical strength. Skin biomechanics is affected by size, shape, number, and arrangement of the epidermal cells, chemical composition and permeability of their cell walls, tissue thickness and turgor pressure (Li et al., 2013; Juxia et al., 2015; Wang et al., 2021). For tomato skin, it has been suggested that round cells provide higher resistance to rupture stress than peel formed by transversely elongated cells (Allende et al., 2004). Similarly for apple, a less cracking-susceptible cultivar had rounded epidermal cells, whereas its counterpart’s epidermal cells were transversely elongated (Juxia et al., 2015). It is worth noting that longitudinally elongated epidermis cells and high cell density may also increase skin resistance to cracking due to increased cell-to-cell contact (Keren-Keiserman et al., 2004). In accordance to the above, the skin of mature pomegranate fruit (24 WAFB) is made up of epidermal cells that are relatively flat and spaced apart (Figure 3C), and it is expected to be less durable against internal pressure; on the other hand, the skin of early fruit (8 WAFB) with two layers of dense and rounded epidermis, and young fruit (11 WAFB) with longitudinally elongated epidermis are expected to be more resistant to cracking (Figure 3; Juxia et al., 2015; Wang et al., 2021).

Furthermore, epidermal cell density was highest in young fruit, decreasing gradually and significantly during fruit development and expansion (Figure 5), thus further weakening the skin. Notably, in pomegranate, the relationship between skin thickness or fruit volume and resistance to fruit cracking was not found to be significant (Saei et al., 2014).

Studies of tomato cuticle have indicated that its mechanical properties are affected by temperature and humidity—both affected cuticle elasticity and stiffness, although RH accounted for much more of the variance than did temperature (Matas et al., 2005). Accordingly, it can be suggested that the heat waves during the period of maximal fruit growth (mid-July to mid-August) plasticize the cuticle, which then cannot resist the growth pressure, resulting in russeting (due to microcracks).

Another factor that is often discussed in relation to skin integrity is mineral content, particularly that of Ca which, along with pectin value, has significant effects on the mechanical properties of the cell membrane in apple (Cybulska et al., 2011). Low-methylated pectin molecules crosslinked with Ca ions make cell walls stiffer, and consequently increase tissue firmness (Saei et al., 2014). In pomegranate, the concentration of most elements [K, N, Ca, P, Mg, and sodium (Na)] has been reported to decrease in the arils and peel during fruit growth and development (Mirdehghan and Rahemi, 2007), and Ca deficiency has been associated with fruit cracking (Singh et al., 2020). In accordance, foliar spray of Ca was reported to significantly reduce pomegranate cracking, however, it occurred when cracking incidence for untreated fruit was very low (Davarpanah et al., 2018; Badawy et al., 2019). In another report, foliar spray of Ca had no effect on the occurrence of pomegranate cracking and russeting (Drogoudi and Pantelidis, 2021). In our study, Ca concentration stayed the same during pomegranate development (Figure 6). The association of Ca level and pomegranate cracking requires further clarification—for example, in Israel, the calcareous soil and high Ca content in the irrigation water are considered to prevent Ca deficiency, however, in some years cracking and russeting disorders are relatively high.

Application of Mg, which also crosslinks with cell wall pectin, has also been shown to reduce pomegranate cracking (Singh et al., 2020); however, in our samples, its level increased in mature fruit (Figure 6). These discrepancies could result from a different sampling method—we sampled only the skin, not the whole peel.

All of the factors discussed so far—epidermis and cuticle properties, mineral composition of the skin, air temperature and humidity—might affect the elasticity/plasticity characteristics of the pomegranate peel. The elasticity of the peel was monitored during 2016 and 2017 in orchards T and S. In 2017, fruit elasticity was reduced as the fruit matured (albeit not significantly), for both the equatorial and longitudinal axes of the fruit—this was more evident in orchard T (Figure 7); in 2016, peel elasticity was maintained until harvest (Supplementary Figure 8). Note that 2017 was characterized by very hot weather, with a high number of days with temperature above 34°C, and a high percentage of small fruit, compared to 2016 with normal temperatures (Table 1). It can be concluded that in 2017, the maturing fruit was small and its skin plasticized, compared to 2016 fruit that was bigger and its skin relatively elastic. Combining these data with those obtained by the Phytech system, the elasticity of the 2016 fruit allowed high-magnitude *mds* values at the time of maximal fruit growth (shrinking is an elastic, reversible adjustment) (Supplementary Figure 2B). We do not have *mds* data for 2017, however, implementing the *mds* of 2015 (Supplementary Figure 2A) when climate conditions were very hot and mature fruit were small, similar to 2017 (Table 1), it can be deduced that small fruit with plasticized skin showed restricted growth rate and low *mds* values (Figure 2D and Supplementary Figure 2A). Accordingly, in 2016, fruit of low quality (mainly cracking and high russeting coverage) amounted to 45.4% of the harvest, whereas in 2015 and 2017, they amounted to only 20–24.4% in each year, although the total yield by weight was lower by 40% than in 2016.

## Concluding Comments

The durability of fruit skin against internal turgor and its resistance to cracking may be determined by epidermal cell structure and environmental conditions, in combination or separately. Rounded and dense cells in the skin of early pomegranate fruit increase its cracking resistance compared to the skin of mature fruit with flat and spacious cells. However, temperate climate at the beginning of the growth period (e.g., in 2016) may support the development of early fruit with elastic skin. The latter, allows for a higher growth rate, the development of big fruit, and maximum daily shrinkage, that together they may lead to pomegranate skin disorders. This is in accordance with a previous report stating that high growth rate weakens the skin and increases fruit susceptibility to cracking (Khadivi-Khub, 2015). On the other hand, restricted growth rate and a low range of daily shrinkage (e.g., in 2015, 2017) may support plasticizing of the mature pomegranate skin, resulting in better peel quality and appearance, probably at the expense of yield. The present work provides insights on pomegranate peel/skin characteristics and demonstrates the implications of global warming on fruit yield and quality.

## Data Availability Statement

The original contributions presented in the study are included in the article/Supplementary Material, further inquiries can be directed to the corresponding author.

## Author Contributions

MJ collected the fruit samples and performed the analyses. ZS provided the Universal Testing Machine for the elasticity studies, and printed the mold used to assemble the peel cuttings with the machine clamps. IG initiated the research project, planned the experiments, carried out the literature review, and wrote most of the manuscript. All authors contributed to the article and approved the submitted version.

## Conflict of Interest

The authors declare that the research was conducted in the absence of any commercial or financial relationships that could be construed as a potential conflict of interest.

## Publisher’s Note

All claims expressed in this article are solely those of the authors and do not necessarily represent those of their affiliated organizations, or those of the publisher, the editors and the reviewers. Any product that may be evaluated in this article, or claim that may be made by its manufacturer, is not guaranteed or endorsed by the publisher.

## Footnotes

1
https://www.phytech.com

2
http://imagej.nih.gov/ij/

3
http://www.jmp.com

## Supplementary Material

Supplementary Figure 1 Percentage of fruit bigger or smaller than 650 g harvested from orchards S and T in the specified years. The percentage is given for each fruit size group (from Table 1 in the main text). Trend lines were added to demonstrate the shift for big fruit in the year 2016 (black) compared to regular size distribution in 2015 and 2017 (red).

Supplementary Figure 2 Maximum daily shrinkage (*mds*) of pomegranate fruit. Data were collected continuously during the growing season using Phytech’s fruit-size sensors, and *mds* was calculated by Phytech’s patented algorithm. **(A)** Data collected during 2015 from orchards H, S, and G, and **(B)** during 2016 from orchards T, S, and G. Orange line marks the end of the first growth phase (see Figure 1B in the main text).

Supplementary Figure 3 Maximum (red) and minimum (blue) temperatures and %RH (green) in orchards S, T, H, and G during June to October of the years 2015 and 2016 as monitored by the Phytech system.

Supplementary Figure 4 Maximum (red) and minimum (blue) temperatures and %RH (green) in the regions of orchard T (upper panel) and orchard S (lower panel) in June–October of the years 2015–2019. Data were provided by the meteorological services of the Ministry of Agriculture and Rural Development, Israel. Temperature range of 30–35°C is highlighted in light orange, and the period of July–August is highlighted in light blue. Note on the *Y*-axis that temperatures in the region of orchard S are higher than in the region of orchard T. Number of days with high temperatures and low humidity during July–August are given in Table 1 in the main text.

Supplementary Figure 5 Association between number of days with temperatures higher than 34°C (blue), or average minimum humidity (%RH; orange) during July–August, and the percentage of fruit bigger than 650 g in the respective years. Data are given for orchards S (2015–2018) and T (2016–2018) based on Table 1 in the main text. Trend lines are given with *R*^2^-values.

Supplementary Figure 6 Pomegranate skin observed by light microscope. Micrographs were taken at 4–6, 8, 11–15, and 24 weeks after full bloom (orchard H, year 2014). Each micrograph was selected from three independent fruit, and represents skin anatomy at the specified sampling time. A yellow line outlines the epidermal layer, and arrows point to the cuticular layer. Bar = 100 μm.

Supplementary Figure 7 Epidermal cell density in pomegranate skin during the growing seasons of 2015 and 2016 in orchards S and T, respectively. The number of epidermal cells in histological sections was counted along a 1-mm line. Values are averages of three replicate fruit ± SE. Data were analyzed for statistical significance by Student’s *t*-test. Mean values with different letters differ significantly at *P* < 0.05.

Supplementary Figure 8 Elasticity of pomegranate peel during 2016 growing season showing data from orchards T and S. Data from each orchard and time point are an average of 5 fruit with ± SE.
