# Supplementary material for: Pomegranate Fruit Growth and Skin Characteristics in Hot and Dry Climate
Source: Front Plant Sci. 2021 Aug 19;12:725479. doi: 10.3389/fpls.2021.725479 (PMC8417319; doi:10.3389/fpls.2021.725479)
Supplement: Supplementary Figure 1 — Percentage of fruit bigger or smaller than 650 g harvested from orchards S and T in the specified years. The percentage is given for each fruit size group (from Table 1 in the main text). Trend lines were added to demonstrate the shift for big fruit in the year 2016 (black) compared to regular size distribution in 2015 and 2017 (red). [file Data_Sheet_1.PDF]

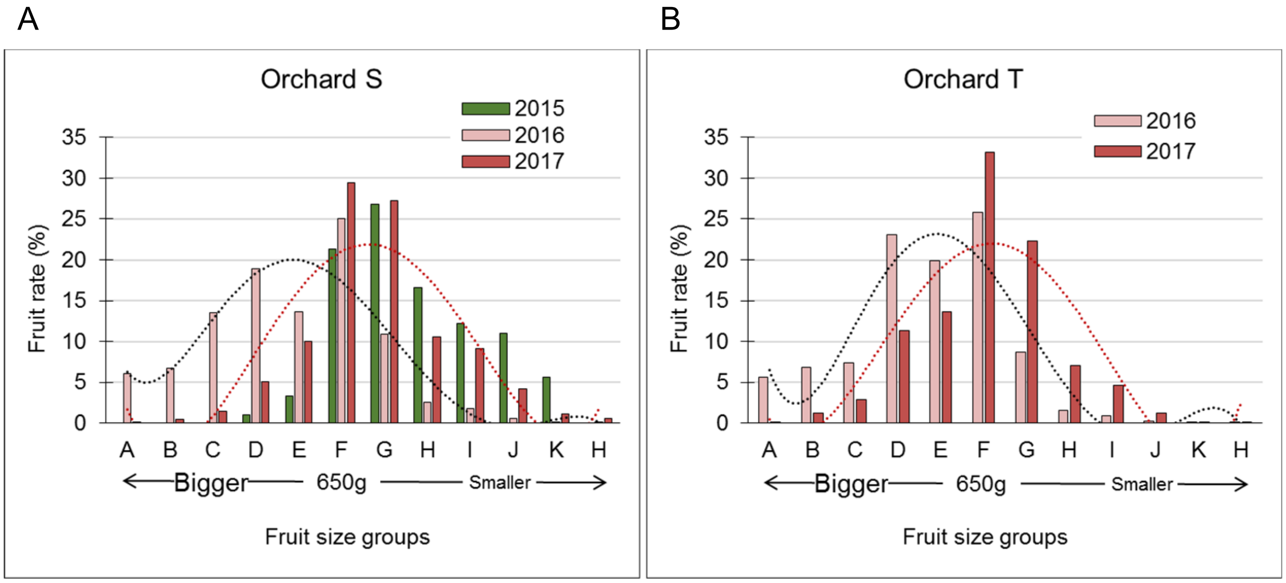

Supplementary Figure 1 Percentage of fruit bigger or smaller than 650 g harvested from orchards S and T in the specified years. The percentage is given for each fruit size group (from Table 1 in the main text). Trend lines were added to demonstrate the shift for big fruit in the year 2016 (black) compared to regular size distribution in 2015 and 2017 (red).

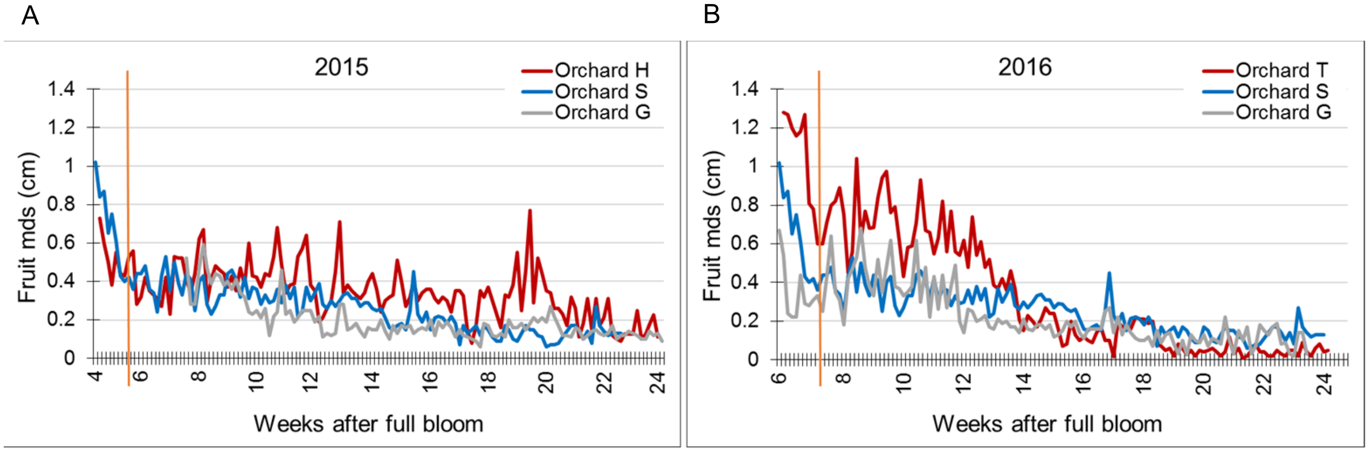

Supplementary Figure 2 Maximum daily shrinkage (*mds*) of pomegranate fruit. Data were collected continuously during the growing season using Phytech's fruit-size sensors, and *mds* was calculated by Phytech's patented algorithm. **(A)** Data collected during 2015 from orchards H, S and G, and **(B)** during 2016 from orchards T, S and G. Orange line marks the end of the first growth phase (see Figure 1B in the main text).

Supplementary Figure 3 Maximum (red) and minimum (blue) temperatures and %RH (green) in orchards S, T, H and G during June to October of the years 2015 and 2016 as monitored by the Phytech system.

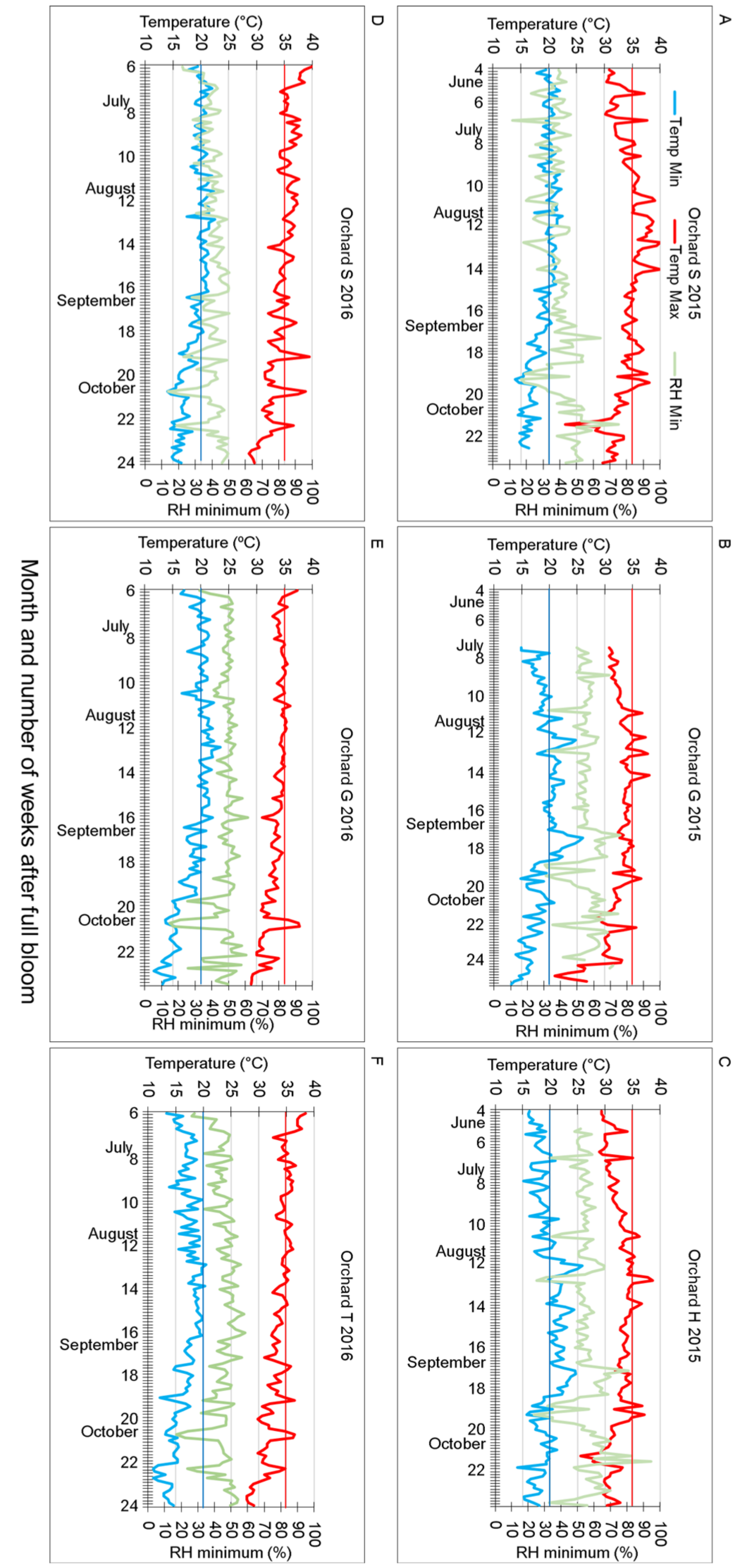

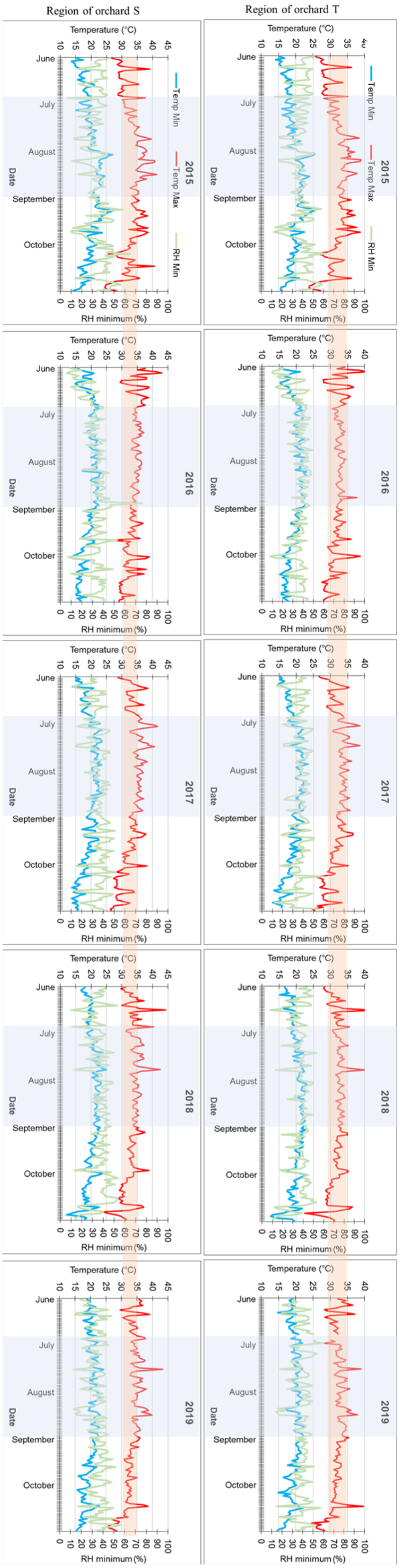

Supplementary Figure 4 Maximum (red) and minimum (blue) temperatures and %RH (green) in the regions of orchard T (upper panel) and orchard S (lower panel) in June to October of the years 2015–2019. Data were provided by the meteorological services of the Ministry of Agriculture and Rural Development, Israel. Temperature range of 30–35°C is highlighted in light orange, and the period of July–August is highlighted in light blue. Note on the Y-axis that temperatures in the region of orchard S are higher than in the region of orchard T. Number of days with high temperatures and low humidity during July–August are given in Table 1 in the main text.

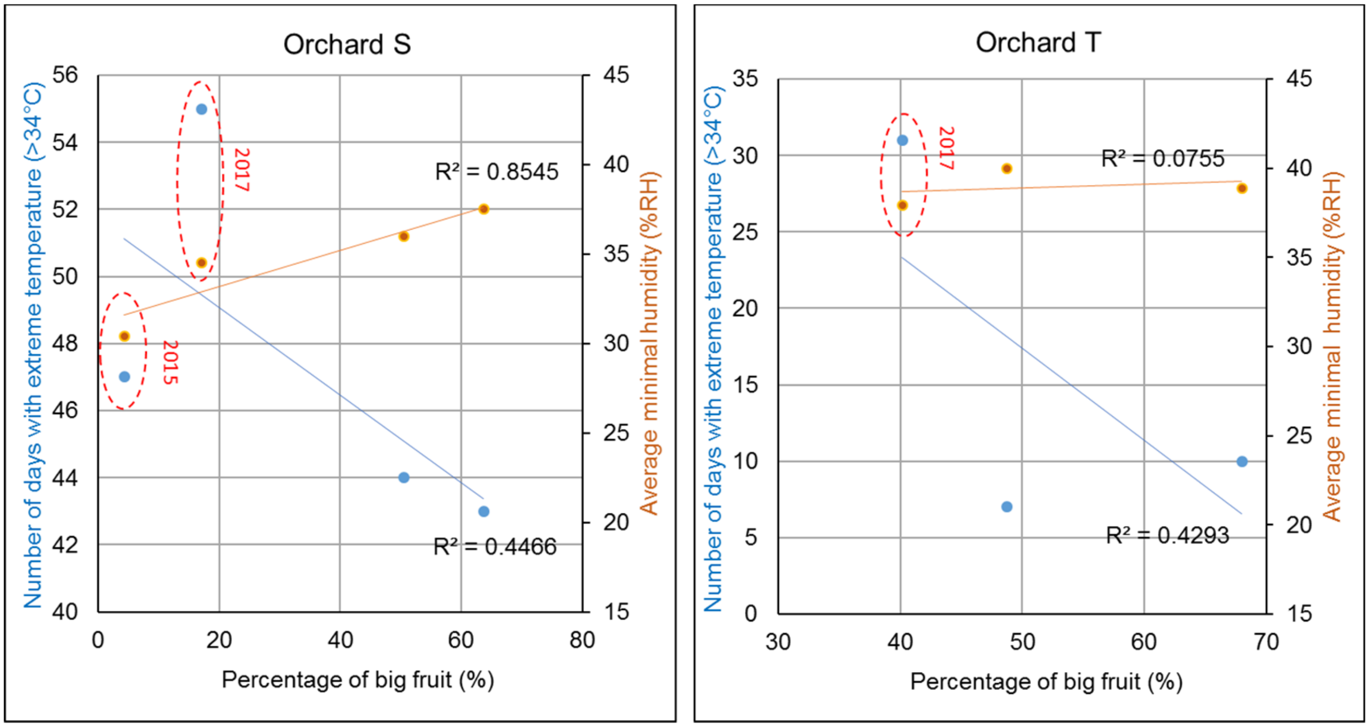

Supplementary Figure 5 Association between number of days with temperatures higher than 34°C (blue), or average minimum humidity (%RH; orange) during July–August, and the percentage of fruit bigger than 650 g in the respective years. Data are given for orchards S (2015–2018) and T (2016-2018) based on Table 1 in the main text. Trend lines are given with R^2 values.

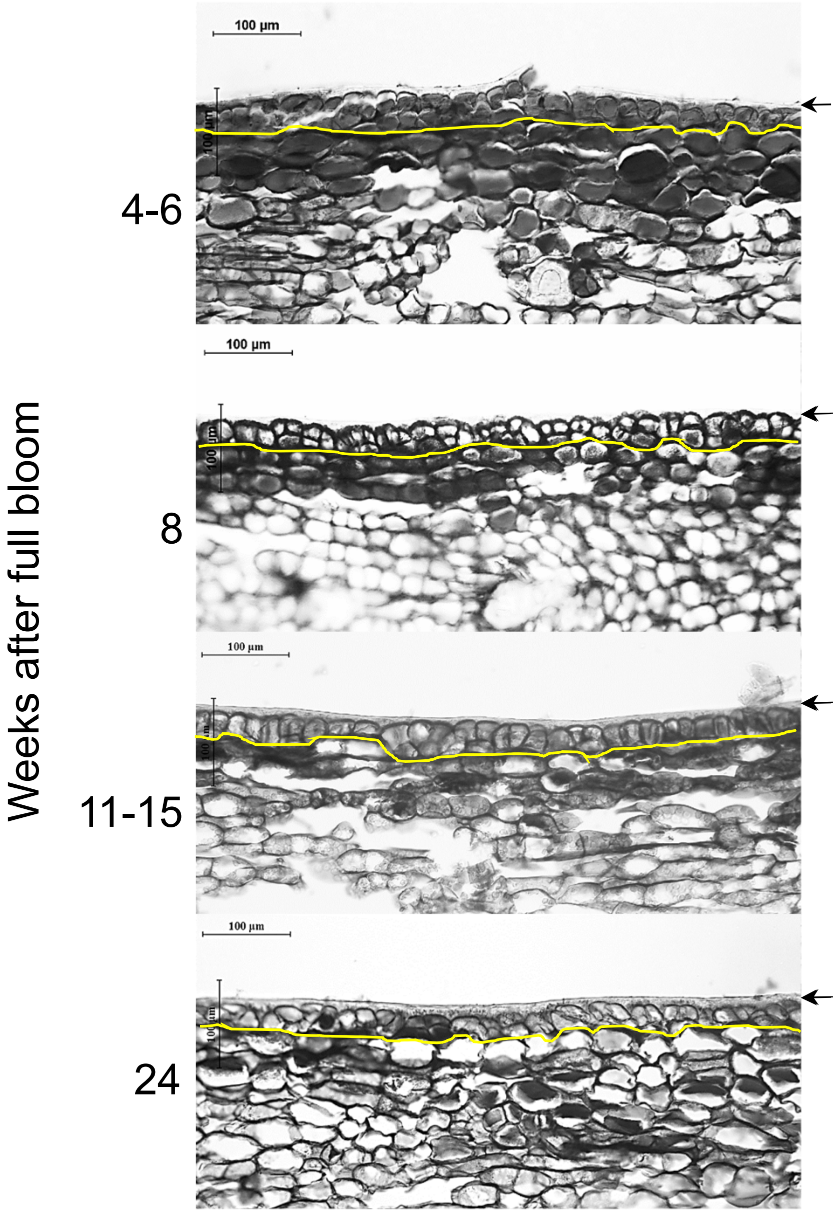

Supplementary Figure 6 Pomegranate skin observed by light microscope. Micrographs were taken at 4–6, 8, 11–15 and 24 weeks after full bloom (orchard H, year 2014). Each micrograph was selected from three independent fruit, and represents skin anatomy at the specified sampling time. A yellow line outlines the epidermal layer, and arrows point to the cuticular layer. Bar = 100 µm.

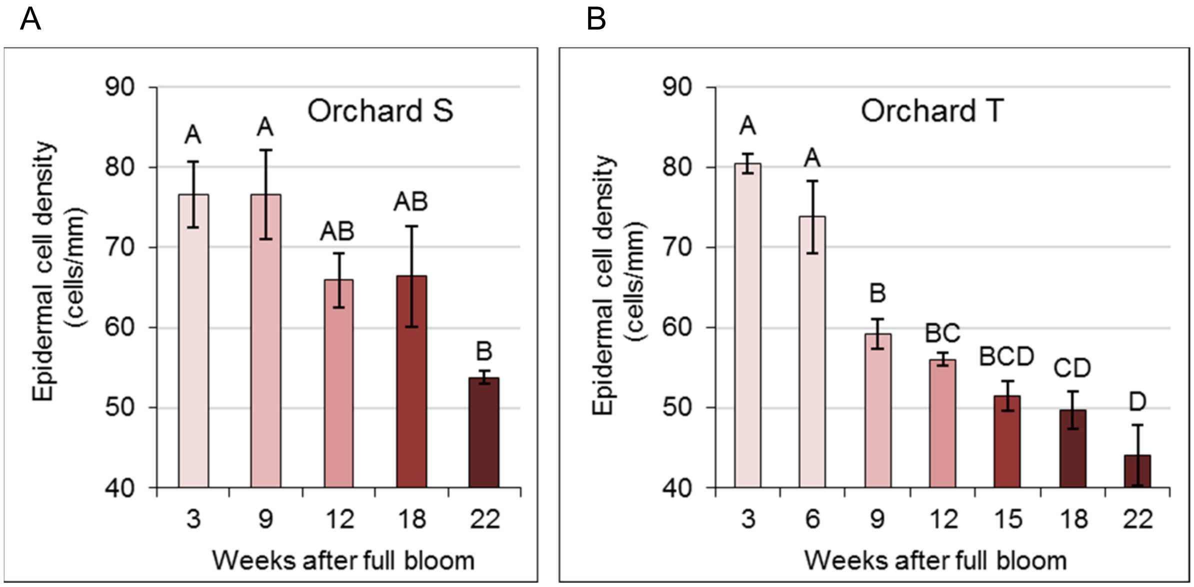

Supplementary Figure 7 Epidermal cell density in pomegranate skin during the growing seasons of 2015 and 2016 in orchards S and T, respectively. The number of epidermal cells in histological sections was counted along a 1-mm line. Values are averages of three replicate fruit ± SE. Data were analyzed for statistical significance by Student's *t*-test. Mean values with different letters differ significantly at $P < 0.05$.

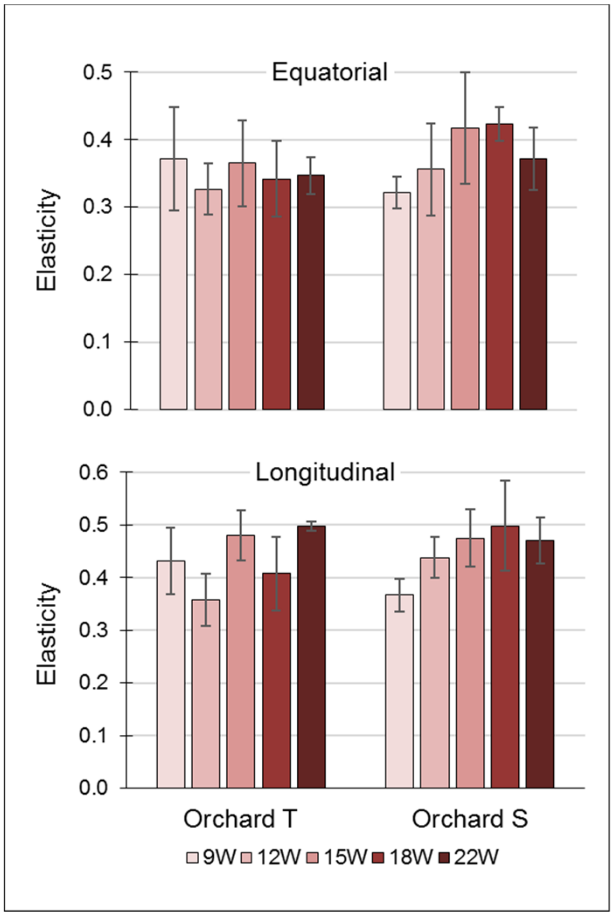

Supplementary Figure 8 Elasticity of pomegranate peel during 2016 growing season showing data from orchards T and S. Data from each orchard and time point are an average of 5 fruit with ±SE.
